# Integrated NMR/MD investigation reveals differences after reweighting in conformational ensembles of the GAAG and GCAA tetraloops

**DOI:** 10.1261/rna.081067.126

**Published:** 2026-08

**Authors:** David Leopold, Andreas Oxenfarth, F. Emil Thomasen, Felix Kümmerer, Robbin Schnieders, György Pinter, Anna Wacker, Hendrik R.A. Jonker, Boris Fürtig, Christian Richter, Kresten Lindorff-Larsen, Harald Schwalbe

**Affiliations:** 1 Institute for Organic Chemistry and Chemical Biology, Center for Biomolecular Magnetic Resonance (BMRZ), Goethe University Frankfurt am Main, Frankfurt/Main 60438, Germany; 2 Structural Biology and NMR Laboratory, Linderstrøm-Lang Centre for Protein Science, Department of Biology, University of Copenhagen, Copenhagen N DK-2200, Denmark

**Keywords:** nuclear magnetic resonances, molecular dynamics, RNA, tetraloop, Bayesian/maximum entropy reweighting

## Abstract

While the GNRA tetraloops are an extensively studied and common RNA motif, their dynamic NMR structures in solution integrating state-of-the-art NMR parameters such as residual dipolar couplings (RDC) and cross correlated relaxation rates (CCR) have previously not been determined. Given their dominant occurrence among tetraloops in the PDB and the advance of experimentally reweighted MD simulations, the present work aims at investigating the entire conformational space of two known tetraloop sequences by an extensive NMR investigation of NOEs, *J*-couplings constants, RDCs and CCRs. As classical structure calculation proved insufficient for the more dynamic tetraloop, we turned to Bayesian/maximum entropy reweighting of molecular simulations using our rich set of experiments. The resulting ensembles were clustered and compared to classically restrained structure calculations, structures from the PDB and models predicted by the prediction algorithms Farfar and AlphaFold 3. Our results show that GNRA-like tetraloops can vary in dynamic sampling of conformational space. They highlight the importance of individual experimental validation of computationally obtained dynamic ensembles and model predictions.

## INTRODUCTION

The number of protein structures deposited in the PDB exceeds the number of RNA structures by more than 25-fold. The lack of structural data on RNAs is daunting, given the increasing awareness of the functional importance of RNAs, especially of noncoding RNAs that make up more than 95% of the human transcriptome (Warner et al. 2018). In the majority of deposited RNA structures, the RNAs are part of an RNA-protein complex, and structures of ribosomes dominate PDB depositions. These structures only partially represent the conformational dynamics of RNA. The underrepresentation of RNA structure results in an insufficient understanding of RNA function as it is often linked to dynamic regions. In fact, these regions provide the necessary flexibility for intra- and intermolecular interaction with *cis*-acting RNA elements (Hermann and Westhof 1999), DNAs (Bacolla et al. 2015), proteins (Dhamotharan et al. 2024) and low molecular weight ligands (Sreeramulu et al. 2021; Vögele et al. 2023; Hymon et al. 2024; Matzel et al. 2024; Ceylan et al. 2025; Toews et al. 2025).

Due to the underrepresentation of RNA structure in the PDB, modern structure prediction programs including Farfar and AlphaFold lack sufficient training data to accurately predict RNA structural ensembles, especially of regions that are not Watson–Crick (WC) base-paired (Bernard et al. 2025). Further, they tend to predict only a single structure, while the true conformational space of an RNA consists of multiple macrostates with associated microstates at room temperature in solution. The microstates as well as the transitions between these microstates are crucial for RNA function (Al-Hashimi and Walter 2008; Ganser et al. 2019). To better understand RNA function, it is therefore vital not only to determine a single RNA structure but also the multitude of accessible conformations at a given temperature. While almost all NMR parameters are sensitive to conformational dynamics, the traditional approach of NMR structure determination heavily relies on proton-proton distance restraints derived from their through-space dipolar interactions at timescales faster than the overall rotational tumbling time. This nuclear Overhauser effect (NOE) has an *r*^−6^ distance dependence. In a multiple state system where two protons are only close to each other in a fraction of the states, distances derived from the observed NOEs are biased toward close distances, since these conformations cause strong NOE peaks. As a consequence, structure determination will yield an energy minimized single structure in agreement with the NOEs and additional dihedral restraints. While conventional structure calculations assume a time averaging regime of nanoseconds and an *r*^−6^ dependence of NOE cross peak intensities, there exists a faster timescale of sub-τ_c_ averaging reaching picosecond timescale, best described by an *r*^−3^ dependence (Vögeli 2014). Implementing such different averaging regimes, however, requires more advanced NOESY analysis and structure calculation protocols. Given our observation here that the NOE proved to be the least sensitive observable to our reweighting of MD (Supplemental Figs. S3, S4), we refrained from incorporating the *r*^−3^ averaging. To determine RNA conformational ensembles, it is therefore essential to determine a comprehensive set of NMR conformation-sensitive parameters in addition to NOEs. Available experimental data include residual dipolar couplings (RDCs) (Zhang et al. 2007; Frank et al. 2009), homo- and heteronuclear *J-*coupling constants (Schwalbe et al. 1993, 1994; Richter et al. 1998; Duchardt et al. 2001) and cross-correlated relaxation rates (CCRs) (Felli et al. 1999; Schwalbe et al. 2002). This wealth of NMR parameters can then be used to reweight and validate independent all-atom molecular dynamics simulations for example by Bayesian/maximum entropy (BME) reweighting (Bottaro et al. 2020a,b; Oxenfarth et al. 2023; Matzel et al. 2025; Wirtz Martin et al. 2025). Half of the observables were therefore used as cross validation to prevent major overfitting issues.

**FIGURE 1.**
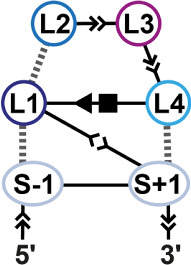
Schematic structure of a tetraloop L1-L4 with a −1 to +1 closing WC base pair, and typical GNRA interactions in the Leontis–Westhof nomenclature.

Here, we benchmark this BME approach by application to two thermodynamically stable, yet dynamic GNRA (N is any nucleotide and R a purine nucleotide) tetraloops. The schematic representation of the typical interaction network stabilizing GNRA tetraloops is given in Figure 1. The four loop nucleotides are marked L1–L4 and the closing base pair nucleotides are denoted *S* − 1 and *S* + 1. Interactions annotated according to the Leontis–Westhof nomenclature (Leontis and Westhof 2001) include the Watson–Crick (WC) base pair between *S* − 1 and *S* + 1 and a sugar-edge-Hoogsteen base pair between L1 and L4. The tetraloop is further characterized by stacking interactions: L1 stacks outward over *S* + 1 and L2, L3, and L4 stack inward in a cascade. In a strict sense, the GAAG tetraloop investigated here is not a GNRA tetraloop. However, it was shown (Okada et al. 2006) that the NMR-derived structure is GNRA-like (Jucker et al. 1996). Additionally, it has been noted that it is not uncommon for tetraloops to adapt structures from other sequence families of tetraloops (Bottaro and Lindorff-Larsen 2017; D'Ascenzo et al. 2017). The importance of predicting structural ensembles is currently actively pursued. It includes the development of a prediction program to sample RNA conformational ensembles and benchmarked their approach for several RNA tetraloops (Williams et al. 2017; Riveros and Yildirim 2024; Herron et al. 2026). The study here and its comparison with the previously determined structural ensembles of the UUCG- (Nozinovic et al. 2010; Bottaro et al. 2020b) and CUUG-tetraloops (Oxenfarth et al. 2023) documents the remarkable plasticity of conformational space even for tetraloops, that form the most stable and most abundant loop architecture elements in RNA sequences. The high stability is thus not driven by a single rigid structure, but the conformational dynamics are in fact inherent to these stable structures, enabling them to act as interaction sites capable of adopting to different interaction partners (DePaul et al. 2010). Further experimental evidence for the higher dynamics of loop nucleotides of these tetraloops can be found in R1ρ measurements as has been performed for the GCAA (Trantírek et al. 2007; Johnson and Hoogstraten 2008), to report on fast dynamics. This work aims to further contribute to the understanding of tetraloop ensembles with at most thorough integrated NMR/MD investigation with two members of the most frequently occurring family of tetraloops.

## RESULTS

**FIGURE 2.**
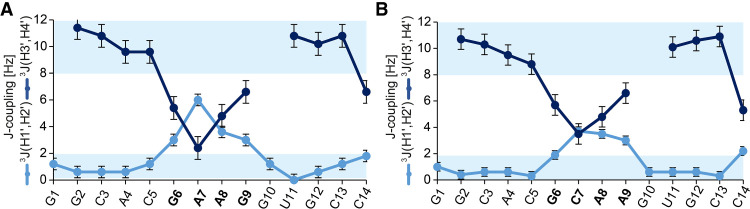
Overview of the determined couplings constants (*A*: GAAG, *B*: GCAA) for all residues (loop residues are bold). (*A* and *B*) ^3^*J*(H1′, H2′) (light blue) and ^3^*J*(H3′, H4′) (dark blue) coupling constants for all residues of GAAG (*A*) and GCAA (*B*). Due to overlap with the water signal, coupling constants for residues G10 could not be determined for both constructs. The typical 3′endo and 2′endo angle range is highlighted by light blue box. The lower box with coupling constants between 0 and 2 Hz indicates the 3′endo range for ^3^*J*(H1′, H2′) and 2′endo range for ^3^*J*(H3′, H4′). The upper box from 8 to 12 Hz indicates the typical 2′endo constants for ^3^*J*(H1′, H2′) and 3′endo constants for ^3^*J*(H3′, H4′). The error for the data is given as standard error.

A broad range of NMR parameters were determined to obtain information on the structure of the two tetraloops. First, we optimized temperature to achieve maximal chemical shift resolution (see Supplemental Material for temperature dependent HSQCs, 298 K for GAAG and 308 K for GCAA). Second, ^3^*J*(H1′,H2′) and ^3^*J*(H3′,H4′) coupling constants were determined (Fig. 2). The pucker conformation of the ribose is typically 3′endo for helical stem nucleotides and can be 2′endo for non-base-paired regions (Varani and Tinoco 1991). The ^3^*J*(H,H) coupling constants of loop nucleotides assume an intermediate value between the range of 2′ and 3′endo. The terminal nucleotides typically show increased flexibility as evident from averaged ^3^*J*-coupling constants. The values of the loop nucleotides agree with dynamic averaging between two or multiple sugar pucker conformations. Apparently, the conformational equilibria are different for each of the four loop nucleotides: L1 deviates only slightly from the typical values for 3′endo, while L2 significantly deviates from 3′endo. However, while all loop residues do not exhibit typical 3′endo *J-*coupling signature, they also do not show coupling constants typical for 2′ endo conformation.

### Extensive NMR experiments yielded high fidelity structural information

**FIGURE 3.**
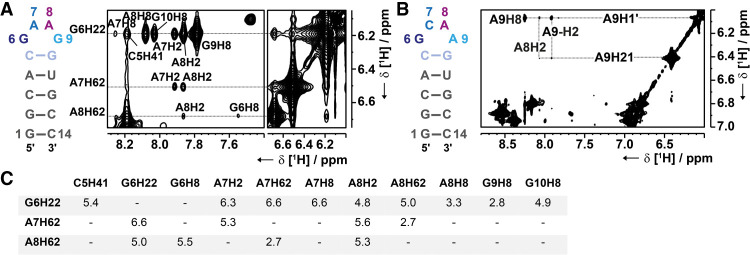
Spectra of the GAAG (*A*) and GCAA (*B*) NOE contacts in loop for the exocyclic amino group. GAAG ^1^H-^1^H-NOESY at 298 K and GCAA at 298 K. The table below (*C*) shows the amino distance contacts to other loop protons according to the iterative process of our ARIA structure calculations, for which NOEs could be detected in the spectra. Values are given in Å.

To proceed with the structural analysis, NOESY spectra were recorded with different mixing times (2× in H_2_O and 3× in D_2_O) and NOE intensities extracted for 295 distances in the GAAG construct and 199 distances in the GCAA construct (Supplemental Table S15) based on the assignment by Schnieders (2020) and Pintér (2020). The NOE intensities were translated to distance restraints using the iterative procedure in ARIA that takes spin diffusion into account. For the GAAG tetraloop, the amino protons of the loop residues G6, A7, and A8 all show cross peaks to aromatic protons in the NOESY spectrum. This NOE network extends to the loop and the closing base pair residues. Especially for residue G6, a large number of nine NOE contacts originating from H22 are observed (Fig. 3). Incorporating the NOE cross peaks at 298 K in the structure calculated by ARIA (RMSD of 0.67 Å) yields ranges for these interactions between 2.7 and 6.6 Å (Fig. 3). In contrast, for the GCAA tetraloop, even when measured at 298 K, no such dense network of NOE cross peaks originating from the amino groups of the loop residues are observable, supporting the hypothesis that the GCAA tetraloops forms a less rigid loop structure. The high flexibility of the GCAA tetraloop observed here by NMR has previously been reported in computational studies (DePaul et al. 2010). Experimental restraints for this study for the amplitude ν^max^ and the angles α, β, γ, ε, and ζ were determined using HCP (Richter et al. 1998), P-FIDS (Schwalbe et al. 1993) experiments for *J*-coupling constant determination and Γ‐HCCH (Felli et al. 1999; Duchardt et al. 2001), Γ-HCP (Richter et al. 2000) and Γ-HCN (Rinnenthal et al. 2007) experiments for the determination of cross-correlated relaxation rates (Supplemental Table S2).

Further restraints for global nucleotide orientation were taken from residual dipolar couplings (RDCs) determined by IPAP spectra of Pf1 phage aligned samples. The alignment tensor was predicted by fitting the RDCs with the structures using PALES (Zweckstetter 2008) and iteratively used in the ARIA structure calculations.

### Structure calculation resulted in unsatisfactory conformations

**FIGURE 4.**
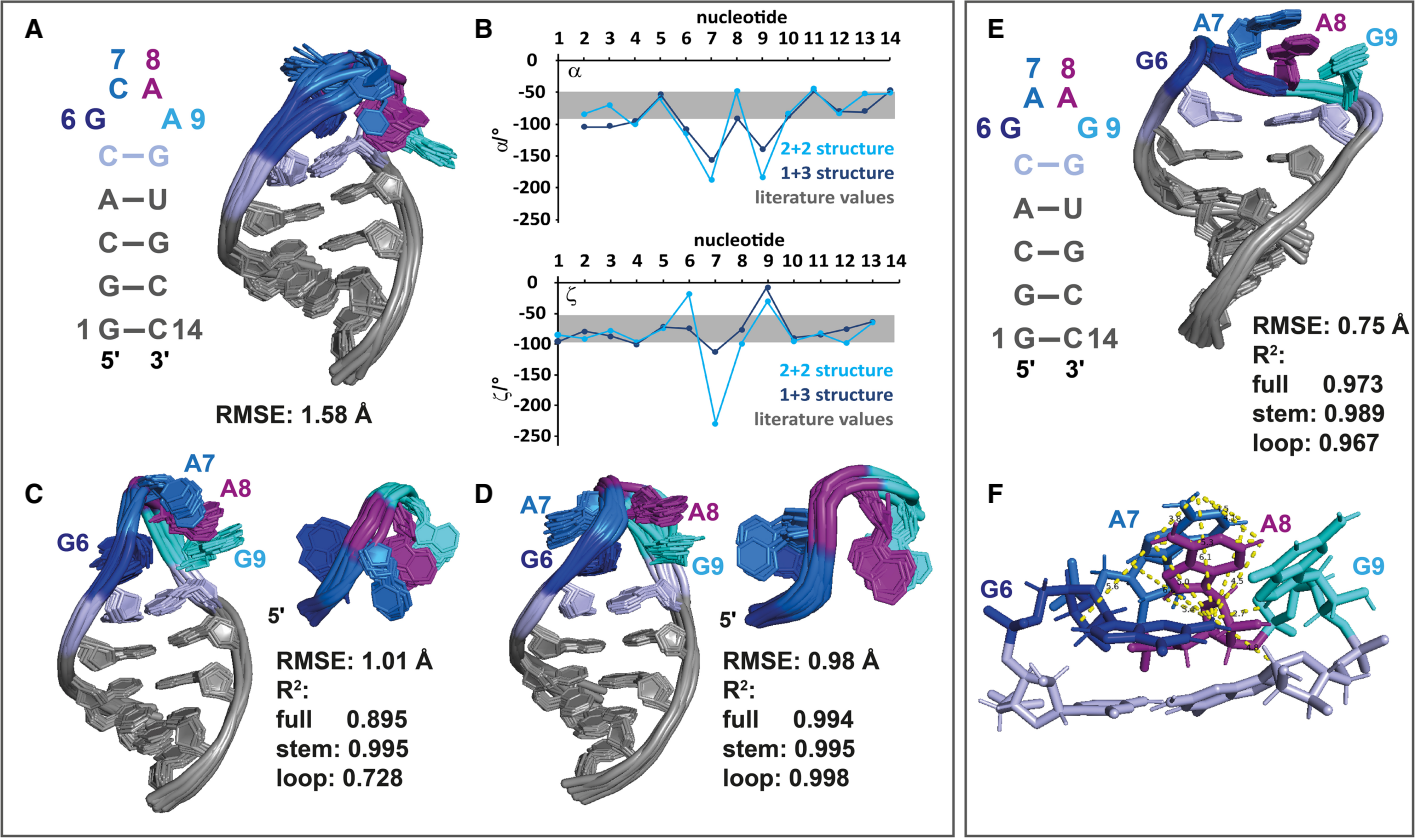
Representation of the two GCAA and GAAG conformations determined by ARIA. The unseparated motifs are shown in *A* and the angular differences of angles α and ζ with a gray bar for the typical 3′endo angles in *B*. The application of two distinct sets of dihedral restraints based on *B* allowed the determination of conformation *C* (also called 1 + 3 structure) with a GNRA-like fold, and conformation *D* (also called 2 + 2 structure) following a AGNN fold and having a higher agreement with the experimentally determined RDCs. Structure *E* shows the single GAAG conformation determined by ARIA. (*F*) Highlights the ^1^H-^1^H-NOE contacts observable from the loop amino protons of GAAG.

Structure determination of the GAAG tetraloop yielded a single structure (PDB 9SY8), which has a GNRA-like tetraloop as previously described by Okada et al. (2006). The structure calculation leading to this single conformation was driven to homogeneity by several NOE signals from the amino protons of nucleotides G6, A7, and A8 in the ^1^H,^1^H-NOESY (Okada et al. 2006; Schnieders 2020), at 298 K indicating a single, stable loop conformation. While ARIA structure calculations for the GAAG tetraloop rapidly converged to a GNRA-like conformation, the initial ARIA calculations using all restraints for the GCAA tetraloop revealed a heterogeneous ensemble consisting of two structural motifs (Fig. 4A). Analysis of the ambiguously restrained dihedral angles using Web 3DNA (Li et al. 2019) allowed further separation of both conformations (Fig. 4B) into two sets of dihedral angles, each corresponding to one conformation. These two sets of dihedral angles were used as constraints for two separate calculations to refine each of the two conformers individually (Fig. 4C,D). The major difference between the conformations is the orientation of the residue C7 (Fig. 4C,D). Furthermore, the orientation of the phosphodiester backbone (see Fig. 4B) differs significantly between the two conformations. We call one conformation the 1 + 3 motif (PDB 9SYD) for its more uneven orientation of loop nucleotides, more akin to the classical GNRA fold with a U-turn fold, while we call the second conformation the 2 + 2 motif (PDB 9SYE), as two pairs of loop nucleotides are oriented on either side of the loop backbone, similar to the AGNN (Wu 2001) tetraloop with a Z-turn fold (D'Ascenzo et al. 2017). Derived RDCs are shown in Supplemental Table S1.

Overall, our NMR data indicate the existence of more than one conformation for the GCAA that are most likely in rapid equilibrium on time scales of milliseconds or faster, as the NMR data are averaged, indicated by the ^3^*J*(H,H) coupling constants of the loop residues that neither show a typical 2′ or 3′ endo conformation (Fig. 2). Strikingly, the RDC data obtained for GCAA are in better agreement with the obtained non-GNRA like fold of 2 + 2. We conclude that the employed ARIA structure calculation protocol is insufficient to capture the complete conformational space of both tetraloops. The discrepancies between experimental restraints and final output was partially too large to trust the structure calculation. Instead, we conducted unrestrained MD simulations that were reweighted by NMR observables to more comprehensively describe the conformational landscape.

### Reweighting of MD with NMR observables yields realistic ensembles

Twenty starting structures for MD simulations of each tetraloop were generated by clustered structures extracted from the PDB based on the conformations of the loop nucleotides with an RMSD cutoff of 1.0 Å and a minimum cluster size of 2 (see Materials and Methods). In the initial stages of preparing MD simulations, we noted that the NMR-observed increased dynamics for the GCAA tetraloop are in line with more diverse conformations observed in the PDB depositions. For the GAAG tetraloop, the clustering procedure resulted in a single cluster containing 19 of the 20 structures (Supplemental Fig. S2A), while for the GCAA tetraloop, three clusters were found, containing nine (Supplemental Fig. S2B), three (Supplemental Fig. S2C) and two structures, respectively (Supplemental Fig. S2D). The remaining six seed structures failed to be clustered. The overwhelming majority of these seed structures represent a GNRA-like structure. Cluster D (Supplemental Fig. S2), however, has a special architecture, with L3 stacking on top of L1 while L2 is positioned above L4, resembling more the typical UNCG motif (Nozinovic et al. 2010).

**FIGURE 5.**
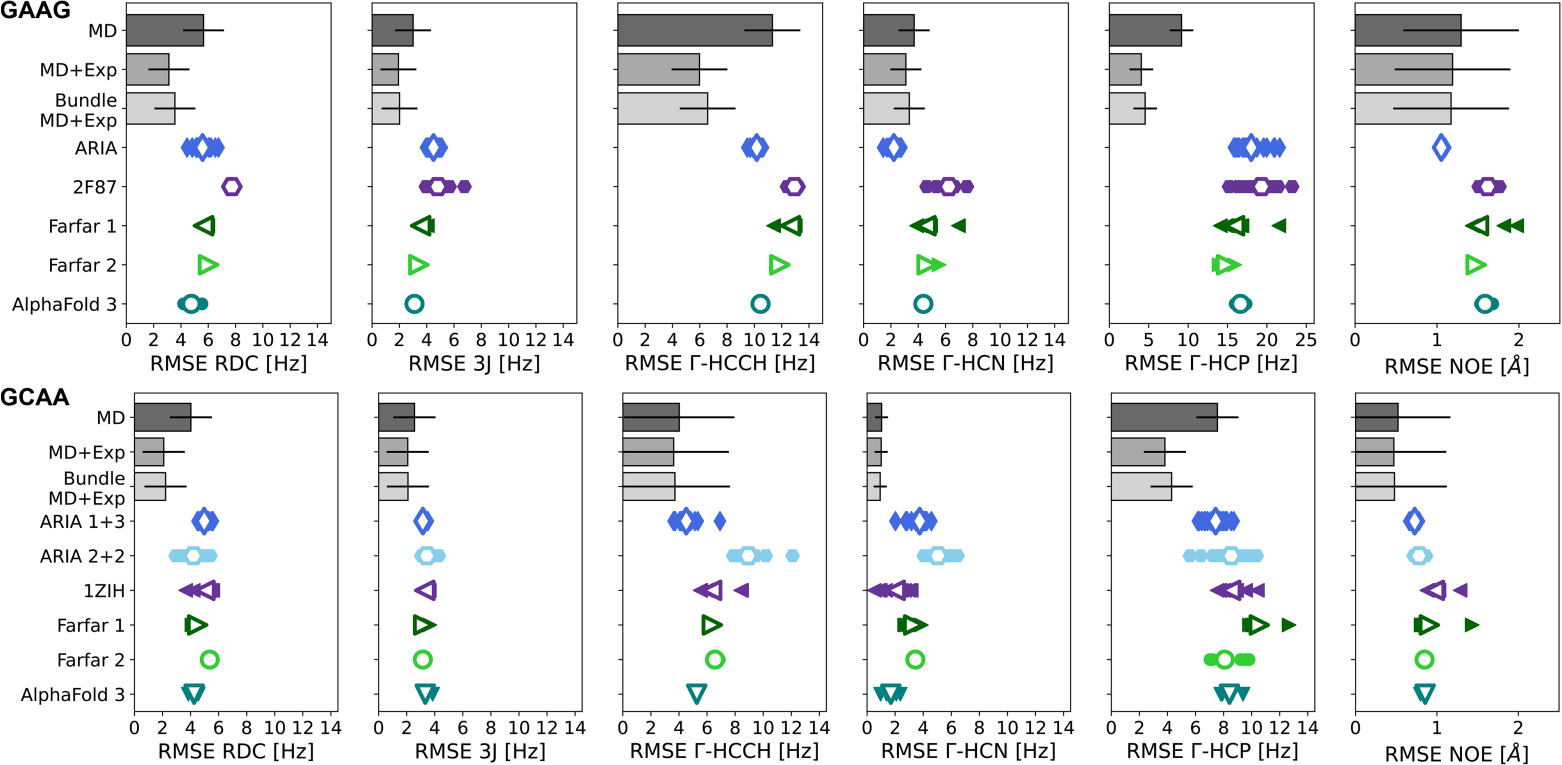
RMSE comparison for all structural ensembles of both tetraloops. The gray bars represent the average of the RMSE difference between all back-calculated data of each MD frame and the experimental determined values with an error range displayed as black string. MD (dark gray) refers to the unweighted simulation, while MD + Exp (gray) the reweighted MD with all frames represents. The Bundle MD + Exp (light gray) is the weighted sub-ensemble of 100 frames. In blue and light blue are shown the structures ARIA (GAAG), ARIA 1 + 3 (GCAA) and ARIA 2 + 2 (GCAA), while the literature structures 2F87 (GAAG) and 1ZIH (GCAA) are displayed in purple. The predicted ensembles of Farfar 1, Farfar 2 and AlphaFold 3 are displayed in different shades of green. As the NMR structure calculation and the predicted ensembles contain a more conceivable number of models, the RMSE difference is displayed for each model. Note: For the Γ-HCP values of GAAG it was necessary to plot a wider *x*-axis range, as the predicted structures featured a worse RMSE than for the other structures.

The simulated MD ensembles (MD) of both tetraloops contain 20,100 frames and NMR observables were back calculated using PALES (RDC), the Barnaba python library (Bottaro et al. 2019) (NOE and ^3^*J*-coupling constants) and a python script for CCRs (Bottaro et al. 2020b). The MD ensembles were reweighted with a Bayesian/maximum entropy (BME) (Bottaro et al. 2020a) algorithm (details in Materials and Methods) based on the RDC, ^3^*J*-coupling constants and Γ-HCP (α, ζ angles), to yield weighted ensembles (MD + Exp). NOEs, Γ-HCCH (ν angles) and Γ-HCN (χ-angle) were used for cross validation. From each of the reweighted ensembles, a sub-ensemble of 100 structures (PDB: 9SYC for GAAG and 9SYF for GCAA) was extracted (Bundle MD + Exp) by random sampling using the weights from the BME to represent the full ensemble by a smaller number of structures. Averages and corresponding RMSEs were calculated for MD + Exp considering the weights of the frames, while uniform weights were used for the sub-ensemble (Bundle MD + Exp). This procedure assumes the time-scale dependence of averaging of the various NMR parameters to be identical. RMSE comparison of the different averages to the experimental data show a reduction of RMSE after the BME procedure (Fig. 5), while cross validation was performed to avoid overfitting (Supplemental Figs. S3, S4). The MD + Exp and Bundle MD + Exp display similar RMSE for all observables, indicating that the sub-ensembles are a good representation of the larger MD + Exp ensemble.

Even before reweighting, the MD simulations generally showed good agreement with the experimental data for both tetraloops. This agreement could be improved through BME reweighting. While the NOE and Γ-HCN agreement improved the least, the most substantial decrease of RMSE is observed for the RDC data, Γ-HCCH (only GAAG) and Γ-HCP values, that report on the phosphodiester backbone conformation. Good agreement is also achieved for the Γ-HCN rates reporting on the glycosidic bond angle χ. Overall, the RMSE of the sub-ensembles is comparable with that of the reweighted ensemble; thus, it is feasible to use the former for clustering and structural analysis.

We further back-calculated the observables of ARIA-calculated ensembles (GAAG = ARIA, GCAA = ARIA 1 + 3 and ARIA 2 + 2) and compared them to the experimental values that gave rise to their restraints (Fig. 5). Additionally, the PDB entries of the tetraloops 2F87 (Okada et al. 2006) for GAAG and the 1ZIH (Jucker et al. 1996) for GCAA were included for the comparison as well as ensembles of lowest energy structures by Farfar 1, Farfar 2, and AlphaFold 3 to compare the results to some currently available 3D structure prediction methods (Fig. 5). Of note, the least fitting observables RMSE-wise of the non-MD ensembles are for the Γ-HCP rates, indicating that the prediction methods are not able to reproduce the experimental CCR data related to the α and ζ angles. This might be caused by the algorithm converging to a low RMSD ensemble with only one conformation being present, while the Γ-HCP rates are better described by a more heterogenous MD ensemble.

Of the three investigated prediction methods, AlphaFold 3 gave rise to structures in closest agreement with the experimental data. This result is perhaps not surprising, since according to the tetraloop investigation of Bottaro and Lindorff-Larsen (2017), the loops with a GNRA-like fold with 10,993 structures made up 57% of all tetraloops in the PDB. The same study, however, found that some loops with GNRA sequence in the PDB (>1%) however, fold in a non GNRA-like way.

### Broader conformational space of MD becomes apparent in the PCA

**FIGURE 6.**
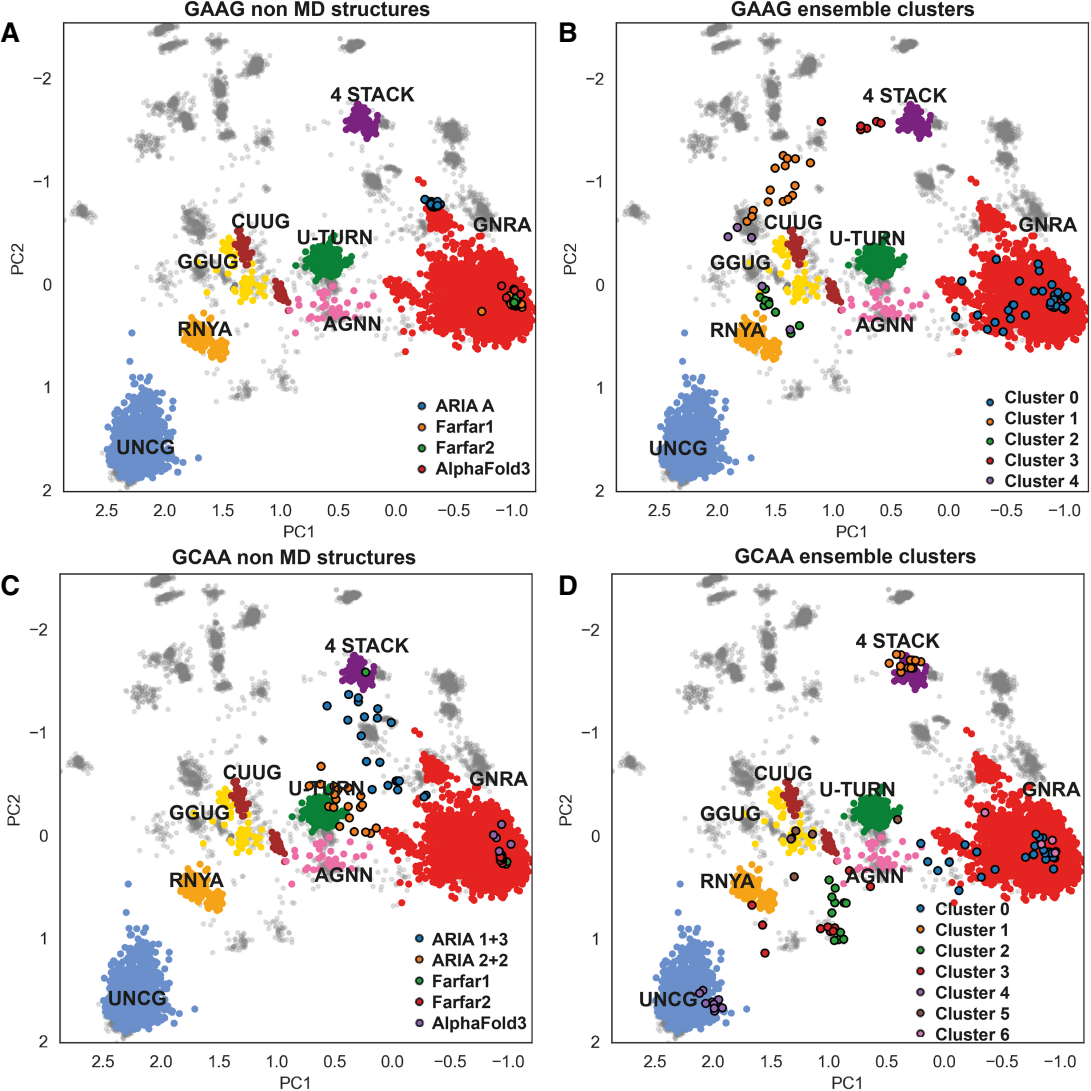
Overview of the PCA plots for GAAG, GCAA, CUUG and UUCG. The PCA plots feature the PDB tetraloops of Bottaro and Lindorff-Larsen (2017) as gray (unclusterd tetraloops) and plainly colored (clustered tetraloops) areas. The data points with a black contour show the different prediction structures (*A* and *C*) and clusters of the Bundle MD + Exp ensembles of GAAG (*B*) and GCAA (*D*).

To determine structural clusters within the ensemble of MD simulation, the “Bundle MD + Exp” was used. A RMSE cutoff of 0.9 Å was chosen with a minimum cluster size of five. Together with a library of tetraloops compiled from the PDB (Bottaro and Lindorff-Larsen 2017), the MD clusters of GAAG and GCAA, the ARIA and predicted structures were subjected to a PCA analysis (Fig. 6) using G-Vectors (Bottaro et al. 2014) based on the approach from Bottaro and Lindorff-Larsen (2017).

It is apparent that the MD clusters of GAAG (Fig. 6B) and GCAA (Fig. 6D) are projected into different areas of the PCA, which is in stark contrast to the predicted and the ARIA structures (Fig. 6A,C), which are projected into or close to the GNRA space of the PCA. Neither Farfar, AlphaFold nor the restrained structure calculation capture the structural diversity of GAAG and GCAA MD clusters. They mainly predict GNRA conformations, except for the ARIA calculations of GCAA that deviate in the PCA from the GNRA space with orientation toward the 4-STACK (Klosterman 2004) and U-TURN (Jucker and Pardi 1995) regions. The typical conformation of these motifs is characterized by different stacking compared to GNRA, with especially G6 in the 4-STACK and A9 in the U-TURN motif deviating from the GNRA conformation. The G6-A9 base pair is not present in both cases (see Supplemental Fig. S1). Thus, the predictions and the “traditional” NMR structure calculation miss capturing parts of the conformational space apparently sampled by the tetraloops.

**FIGURE 7.**
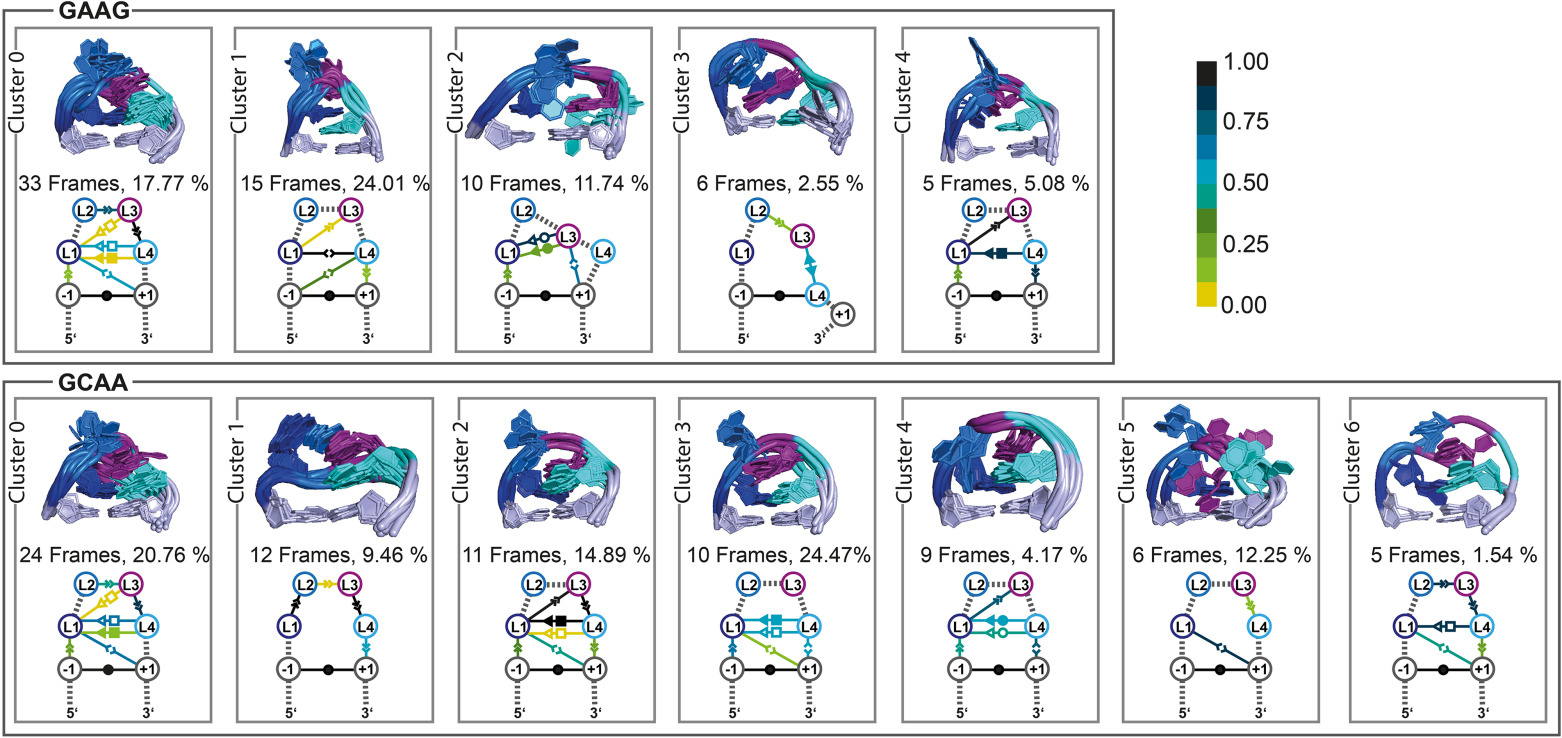
Three-dimensional and schematic 2D structures of the GAAG and GCAA clusters of the sub ensembles Bundle MD + Exp (only the loop and closing base pair residues are shown). In the schematic 2D representation of the clusters, the base interactions found by Barnaba are depicted in a color scheme based on the population of those interactions within the cluster.

Stacking and base-pairing in each cluster were analyzed using the Barnaba python library (Bottaro et al. 2019). Figure 7 shows the clusters ordered by their number of contributing frames. This order deviates from the weighting order of the clusters, which is indicated as percentage for each cluster.

Of the 100 frames of the GAAG sub ensemble, five clusters were identified. Together these clusters are composed of 69 frames and 61.15% weighting contribution of the GAAG “Bundle MD + Exp.” Cluster 0 is attributed to the GNRA conformation, based on the PCA results, while the other clusters do not fall into a specific tetraloop category. Cluster 5 is, however, similar to UNCG-like fold and Cluster 2 features several interaction motifs similar to the U-Turn (Bottaro and Lindorff-Larsen 2017) motif (Fig. 7; Supplemental Fig. S1). GAAG's most highly weighted cluster 1 with 24.0% contribution to the “Bundle MD + Exp” displays a structure quite different from the conventional GNRA motif with a strong base-pairing interaction of G6 and A8 and a bulged out G9. A strong stacking interaction of G6 and A8 can also be observed in cluster 4. Cluster 3 is interesting due to a bulged-out G10, where C5 and G9 form a “closing base pair.”

The GCAA clusters can be categorized into four macrostates: GNRA, UNCG-like, 4-Stack, and CUUG-like, with some clusters representing microstates that have not fully formed a certain macrostate. A more detailed analysis of base-pairing and stacking interactions of the clusters was performed with Barnaba and is detailed in the Supplemental Material, also including the structural features of the two second-highest weighted frames of each MD simulation (Fig. 7; Supplemental Fig. S9). Analysis of all loop residue backbone dihedral angles is shown in Supplemental Figures S10–S25. The populations of the base-pairing and -stacking interactions (Supplemental Fig. S7 [GAAG] and Supplemental Fig. S8 [GCAA]) show the deviation from the GRNA motif of the MD ensemble. This deviation is more apparent after reweighting. Interactions, characteristic for GNRA like G6–G9 base-pairing or A7–A8 stacking, are less populated in the initial MD and significantly less populated after the reweighting (MD + Exp). Interactions observable in the non GNRA clusters, which are not present in the prediction and ARIA ensembles, are more populated in the MD + Exp ensemble. For most interactions the populations of MD + Exp and Bundle MD + Exp are very similar. In some cases, their populations differ, like in the case of the A8–G10 stacking of GAAG, where the sub ensemble did not capture the MD + Exp ensemble perfectly.

**FIGURE 8.**
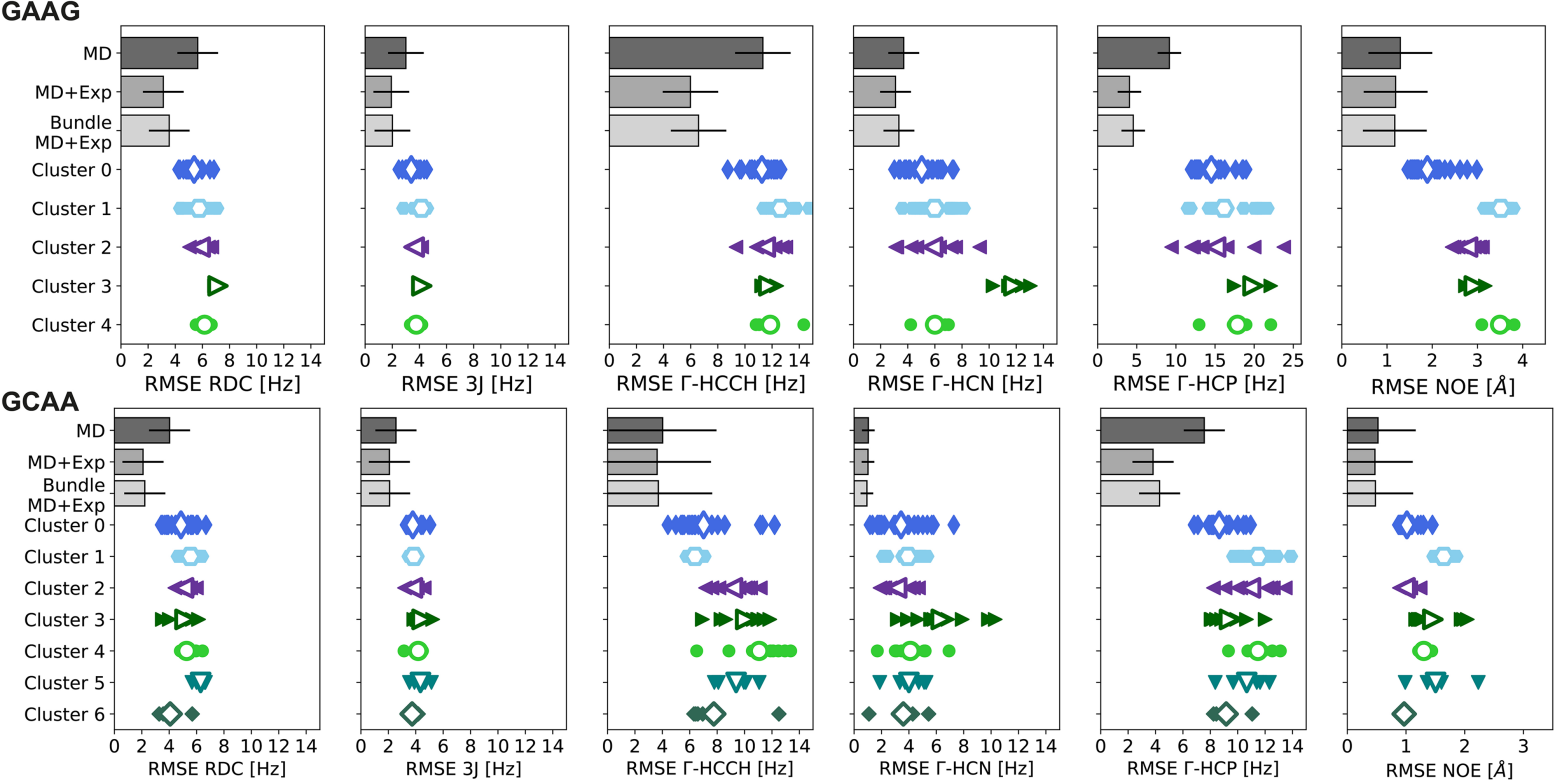
RMSE comparison between the clusters and the MD ensembles for both tetraloops. The gray bars represent the average of the RMSE difference between all back-calculated data of each MD frame and the experimental determined values with an error range displayed as black string. MD (dark gray) refers to the unweighted simulation, while MD + Exp (gray) represents the reweighted MD with all frames. The Bundle MD + Exp (light gray) is the weighted sub-ensemble of 100 frames. The colored data points represent the different clusters extracted from the Bundle MD + Exp (light gray). Note that for the CCRs determined by the Γ-HCP experiment for GAAG, the RMSE scale had to be increased to 25 Hz.

To determine whether any of the individual clusters extracted from MD-Exp, fit the experimental data better than others, their observables were back-calculated and compared to the experimental values (Fig. 8). Our results indicate that neither of the clusters can reproduce the experimental data on its own and only by combining and averaging the observables of a sufficiently diverse ensemble is a low RMSE obtained.

## DISCUSSION

This work encompasses the investigation of RNA tetraloops with NMR spectroscopy as the method of choice to reliably observe the conformational heterogeneity of RNA molecules. We measured NOEs, *J*-coupling constants, RDCs and CCRs, which we used to derive an MD ensemble reweighted and validated by this extensive set of NMR parameters. The obtained structural model enables us to describe the dynamic loop architecture featuring numerous noncanonical base pair interactions at atomic resolution. Given that proteins can read out subtle structural differences as basis for recognition and regulation, such level of detail is required to grasp the structure-function relationship linked to RNA (Varani and Nagai 1998; Jones 2001).

Our work is motivated by providing answers to the question how to best represent the “true” structure of tetraloops at ambient temperature under near physiological conditions. Such a holistic structure model is important as dynamic regions of RNAs are important for the plethora of RNA function. We analyzed our MD-Exp structural ensembles by PCA, demonstrating the sampling of a substantial larger conformational space than apparent by the PDB structures (Jucker et al. 1996; Okada et al. 2006). Our work further solidifies the value of combining MD simulations with NMR data and provides insights into the relationship of previously unreported or rarely reported loop architectures for the investigated tetraloops. Specifically, we report several states adopted in solution that do not conform to the GNRA structure (Heus and Pardi 1991). According to our findings, the GCAA and GAAG tetraloop exchange between several folds of which some display a U-turn (major turn between residue L1 and L2) and some a Z-turn (major turn between L2 and L3) (D'Ascenzo et al. 2017). In contrast, the more stable UUCG and CUUG tetraloops occupy several folds, but exclusively with Z-turns (D'Ascenzo et al. 2017). Most strikingly, Z-turn folded motifs are not predicted by Farfar and AlphaFold and restrained structure calculations struggle or fail to find these folds due to the overemphasis on a single lowest energy structure or the scarcity of non GNRA motifs (Bottaro and Lindorff-Larsen 2017) on the structural databases for training. While the exact implications of these different tetraloop folds need to be further studied, a reasonable idea is that the more dynamic nature of these loops allows for easier access into rigid binding domains, with the respective tetraloop binding motif folding only into its final state posterior to binding initiation. The more exposed nucleobases of some of these motifs might work as binding initiators, “scanning” for possible hydrophobic pockets. The fact that folds like 4-STACK and UNCG-like which are known to bind RNA or proteins (Banks and Linial 2000; Klosterman 2004; Huang et al. 2010) appear in solution for a tetraloop with a major GNRA-like fold, indicates that the binding capabilities of some GNRA-like tetraloops might be more flexible as one would anticipate. Whether such a tetraloop like the GCAA can bind different interaction sites with different folds or whether the major fold of GNRA impairs this needs to be further studied. We are certain our experimental data will prove helpful in the ongoing assessment of RNA folding predictions.

## MATERIALS AND METHODS

### NMR sample preparation

Natural abundance and ^13^C,^15^N-labeled RNA 14mer samples were produced using T7-polymerase in vitro transcription. The plasmid contained an *EcoR1* sequence followed by a *T7*-promoter and the respective 14mer sequence followed by an *HDV* ribozyme for homogeneous 3′ terminus and *Smal* sequence for linearization of the plasmid. Amplification of the plasmid was performed with the DH5α *Escherichia coli* strain and purified by Gigaprep (Qiagen). The preparative transcription was performed with the *P226L* mutant of the T7-polymerase for 16 h at 37°C. RNA was precipitated using pure isopropanol. Precipitated RNA was loaded on a Midigel to separate the *HDV* from the target RNA. The target RNA was eluted from the cut gel band with 3 m NaOAc aqueous solution and precipitated with pure isopropanol. Further purification by preparative HPLC using a Kromasil RP18 column was performed and the target RNA predicated with LiClO_4_ (2% w/v in acetone). Buffer exchange to NMR buffer (50 mM potassium phosphate, pH 6.4) was achieved via centrifugal concentrators with a 2 kDa MWCO membrane. The sample was heated at 95°C for 1 min and slowly cooled to room temperature for 5 min for folding. The final sample volume contained 280 µL buffer with a DSS concentration of 50 µM as reference. RNA concentrations of different samples ranged from 0.48 to 1.2 mM. The RNA was dissolved either in D_2_O or in H_2_O containing 5% D_2_O. After sample preparation the 5′-end of the 14mer RNA should carry a triphosphate and the 3′-end a 2′,3′ cyclic phosphate group (Schurer et al. 2002).

For RDC measurements, samples of 200 µM for the GAAG and GCAA were prepared with and without 20 mg/mL of Pf1 phage coat protein (ASLA Biotech AB, RNase, and protease free) in a 10 mM KP_i_ D_2_O buffer solution. The deuterium splitting in an 800 MHz spectrometer in the aligned GAAG sample was 28.54 Hz at 298 K and 29.79 Hz in the aligned GCAA sample at 308 K.

### NMR experiments

NMR experiments were carried out on Bruker spectrometers operating at ^1^H frequencies of 600, 700, 800, 900, and 950 MHz equipped with cryogenic probes and either AVIII, AVIIIHD or AV NEO consoles. Measurement temperatures were optimized for the least peak overlap to be 298 K for GAAG and to be 308 K for GCAA.

Processing and analysis of the NMR data were conducted using the software TopSpin 4.2.0 (Bruker BioSpin). Internal DSS standards were used for chemical shift referencing with the Wishart module (Wishart et al. 1995) for nonproton resonances. The spectral data can be accessed here (https://gude.uni-frankfurt.de/handle/gude/776). Resonance assignment and intensity determination was performed with the NMRFAM-Sparky (Lee et al. 2015) program.

### Structure prediction using Farfar and AlphaFold 3

For Farfar 1 and Farfar 2 structure prediction, the ROSIE website was used (Lyskov et al. 2013). Over 10,000 Monte Carlo cycles, 2000 structures were calculated; the 20 lowest energy structures derived and their observables back-calculated for comparison.

For structure prediction of both tetraloops with AlphaFold 3, the official webserver was used to generate bundles of the five lowest energy structures (Watkins et al. 2018). The Molprobity server (Williams et al. 2018) was used for hydrogen addition to the AlphaFold 3 files using the “no flips” and “nuclear x-H” option.

### ARIA structure calculation

RNA structure calculations with ARIA CNS 1.1 (Brunger 2007) were carried out with an in-house setup using the nucleic acid force field (dna-rna-allatom-hj, https://gude.uni-frankfurt.de/handle/gude/776) (Nozinovic et al. 2010). Modifications included optimized potentials for liquid simulations (OPLS) charges and nonbonded parameters (Linge et al. 2001; Rieping et al. 2007). The iterative procedure was applied as standard for the NOE distance calibration, with the use of floating chirality assignment and spin diffusion correction. For spectra measured in H_2_O, a separate NOE distance calibration was used for the protons that are prone to exchange with water. One hundred starting structures were generated in the initial seven iterations with 20 structures being used for the following iteration. In the eighth iteration, 200 structures were generated, from which 20 structures were used for the final explicit water refinement (Linge et al. 2003). After each iteration, the NOE distance restraints were recalibrated. The calculation of each iteration was a four-staged simulated annealing (SA) procedure with torsion angle dynamics (TAD). From an initial high temperature stage of 10,000 steps at 10,000 K a refinement stage was applied at 2000 K for 8000 steps, followed by two cooling down stages to 1000 K in 20,000 and finally to 50 K in 15,000 steps. During the SA protocol, the force constant of the NOE restraints was gradually increased from 10 to 50 kcal mol^−1^ Å^−1^. The RDC alignment tensor (axial and rhombic components) was calculated from the experimental data (by best-fit and back-calculation) using the Pales software (Zweckstetter 2008) and the restraints were applied in the ARIA structure calculation starting from the fifth iteration.

Distance restraints were calculated by ARIA, using five ^1^H,^1^H-NOESY experiments for each RNA construct of varying mixing time and solvent. Three spectra were measured in D_2_O with mixing times of 50, 100, 150 msec for the GAAG tetraloop and 100, 150, 250 msec for GCAA tetraloop and two spectra in H_2_O with 5% D_2_O with mixing times of 100 and 150 msec for GAAG tetraloop and 150 and 250 msec for GCAA tetraloop. Further restraints included weak base pair and planarity restraints for the stem base pair. Dihedral restraints were derived from ^3^*J* coupling and cross correlated relaxation (CCR) data and were applied during the simulated annealing with increasing the force constant from 5 to 200 kcal mol^−1^ rad^−2^. When the experimental data indicated two possible dihedral angles, an ambiguous restraint was set, allowing both angle conformations during the calculation, with weaker penalties for the shorter region between these angles, making conformational shifts possible even in later annealing stages. As validation of the final structure calculations and as aid in the reiteration of the ARIA process, the RDCs were subjected to back-calculation and the alignment tensor re-evaluated with PALES. The agreement to this value is given by the correlation coefficient *R*^2^. If a structure ensemble revealed only a low RMSD ≤1 Å, the averaged *R*^2^ was determined for the ensemble.

### MD simulations

MD simulations were performed as described previously (Oxenfarth et al. 2023). For the seeds of the MD simulations, the protein data bank (PDB) was searched for all deposits containing the respective tetraloop sequence with a closing CG base pair (5′-ncGCAAgn-3′ or 5′-ncGAAGgn-3′). A complete-linkage hierarchical clustering was performed for each set of PDB deposits using the eRMSD for distance measurement (Bottaro et al. 2014). The final number of clusters was set to 20 to allow for a sufficiently large set of seed structures for a diverse set of MD simulations. Due to lack of diversity within the PDB, the GAAG however was sampled from a set of seed structures of low RMSE (see Results; Supplemental Fig. S2). The stem of G1-C5 and G10-C14 was constructed as an ideal A-form helix using a python scrip (https://github.com/sbottaro/build_aform). The 20 loop constructs were linked to the stem construct by aligning the heavy atoms in residues 5 and 10 as well as the heavy backbone atoms in residues 4 and 11.

The constructs were subjected to modeling with the Amber ff99SB force field with parmbsc0 and *X*_*OL3*_ corrections (Pérez et al. 2007). The RNA was minimized in vacuum for up to 50,000 steps of steepest descent to be placed in a dodecahedron with a padding of 1.4 nm between RNA and dodecahedral outlier. A total of 4300 OPC water molecules (Izadi et al. 2014) were added as solvent, and the system was minimized for up to 25,000 steps. The negative charge caused by the RNA was neutralized by addition of K^+^ ions. To obtain an ion concentration of 10 mM, further K^+^/Cl^−^ ions were added and the system minimized for up to 25,000 steps. The system was equilibrated in the NVT (constancy of number of particles, volume and temperature of system) ensemble for 200 psec by using the velocity rescaling thermostat (Bussi et al. 2007) with a timescale of 1 psec and a reference temperature of 308 K. Harmonic position restraints of the heavy RNA atoms were performed with a force constant of 1000 kJ mol^−1^ nm^−2^. This was followed by equilibration in the NPT (constant number of particles, pressure and temperature of system) for 200 psec each using the Parrinello-Rahman barostat for pressure coupling, while the force constants of positional restraints were gradually released to 0 kJ mol^−1^ nm^−2^. During the NPT simulation a reference pressure 1 bar was used, time scale of 2 psec and an isotropic compressibility of 4.5 × 10^−5^ bar^−1^. The final simulations were performed in the NPT ensemble for 200 nsec for each of the 100 simulations. Equations of motion were integrated with the leapfrog algorithm, and a cutoff of 1.4 nm was used for van der Waals and Coulomb interactions. Long-range electrostatics were incorporated using the particle-mesh Ewald summation (Essmann et al. 1995) with fourth order cubic interpolation and a 0.16 nm Fourier grid spacing. Every of the 20 structures was used as starting points for five independent, 200 nsec simulations, leading to 100 simulations that were pooled together (totaling 20 msec) and used for analysis by extracting a frame for every 1 nsec of runtime.

### Reweighting and MD simulation analysis

For the reweighting procedure, the experimental data of NOE contacts, *J*-coupling constants, RDCs, and CCRs were converted to a uniform format based on the procedures described in the GitHub library (https://github.com/KULL-Centre/_2023_oxenfarth_CUUG), modifying it to fit the respective tetraloops. For the analysis of eRMSD, *J*-coupling constants, dihedral angles and base pair interactions, the Barnaba python (Bottaro et al. 2019) library was used. CCRs were handled separately based on the experiment (Γ-HCN, Γ-HCCH, Γ-HCP) used to determine them and were handled using a python script (https://github.com/sbottaro/CCRR), while for the simulation averages of the NOE distances, the compute_distance module of the MDTraj python library (McGibbon et al. 2015) was used with an *r*^−6^ averaging. RDCs were calculated with the PALES (Zweckstetter 2008) software: The alignment tensor prediction was performed with Rod LC model (pf1) with a crystal concentration of 0.05 mg mL^−1^. Comparison of experimentally determined and simulation-based calculated RDCs was done after rescaling the calculated values using a linear regression model considering all 14 residues.

For the BME reweighting (Bottaro et al. 2020a), several combinations of experimental data were tested in fivefold cross-validation; however, the combination of RDC, Γ-HCP CCR and ^3^*J* couplings proved most suitable for both tetraloops using a hyperparameter θ of 16. The experimental data not used (NOE, Γ-HCCH, Γ-HCN) as fitting parameters were subsequently used for cross validation. Clustering and base interaction analysis were done with a sub-ensemble of 100 frames, extracted from the 20,100 available frames using the python random.choice module under consideration of the weighting of the frames, with the replacement = False setting. The clustering was based on the scripts used for the CUUG tetraloop (Oxenfarth et al. 2023) using a quality threshold algorithm (Heyer et al. 1999) with an eRMSD cutoff of 0.9 Å and a minimum cluster size of 5. Visualization of the analysis and structures is done using Python Matplotlib (Hunter 2007) and Pymol (The PyMOL Molecular Graphics System, Schrödinger LLC). PCA was performed using an adapted version of the code in https://github.com/sbottaro/RNA_TETRALOOPS.

## DATA DEPOSITION

NMR raw data and python scripts for their analysis are available at the Goethe University Frankfurt Data Repository (https://gude.uni-frankfurt.de/handle/gude/776). The NMR ARIA structures are available as 9SY8 (GAAG), 9SYE (GCAA 2 + 2) and 9SYD (GCAA 1 + 3). The MD sub-ensembles are available as 9SYC (GAAG) and 9SYF (GCAA). The scripts for the observable back-calculation, BME reweighting, clustering and data analysis are available at https://github.com/KULL-Centre/_2026_leopold_GNRA/.

## SUPPLEMENTAL MATERIAL

Supplemental material is available for this article.

## Figures and Tables

**David Leopold:**
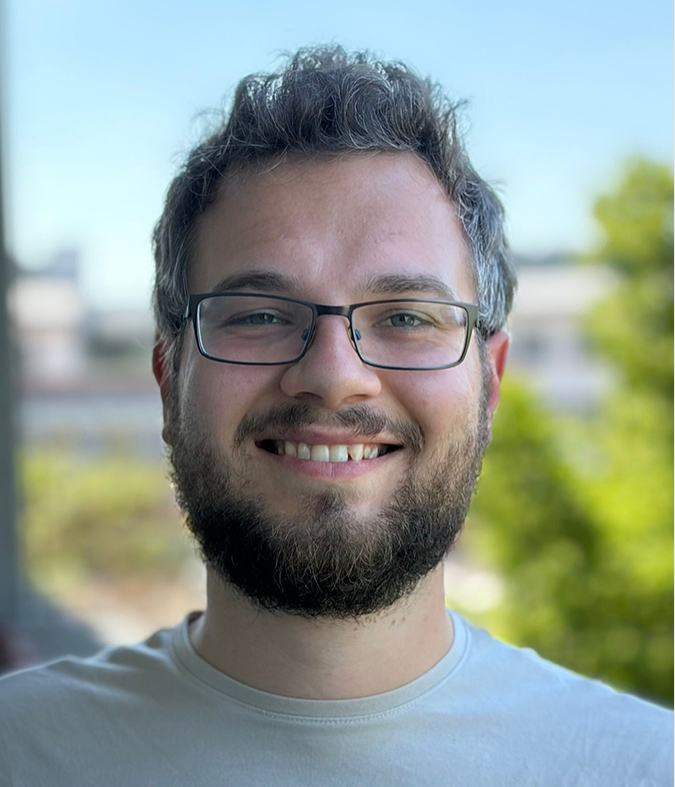


## MEET THE FIRST AUTHOR

***Meet the First Author(s)* is an editorial feature within *RNA*, in which the first author(s) of research-based papers in each issue have the opportunity to introduce themselves and their work to readers of *RNA* and the RNA research community. David Leopold is the first author of this paper, “Integrated NMR/MD investigation reveals differences after reweighting in conformational ensembles of GAAG and GCAA tetraloops.” David is a third year PhD student in the Schwalbe Lab in the Department of Organic Chemistry and Chemical Biology at Goethe University, Frankfurt am Main, Germany. The focus of his research is on the dynamics of RNA and its minor states.**

**What are the major results described in your paper and how do they impact this branch of the field?**

We determined an extensive set of NMR observables for two RNA hairpins, capped by the tetraloops GAAG and GCAA, which are forming GNRA motifs according to current literature. Previous studies relied on NOEs for distance and *J*-couplings for dihedral restraints. We extended the set of used *J*-couplings and measured residual dipolar couplings for long-distance restraints and cross correlated relaxation rates for a more complete set of dihedral restraints. The additional insights, however, hinted to several conformations in the loop region of the RNAs, so we performed molecular dynamic simulations and reweighted the resulting frame composition based on our NMR observables to obtain a more faithful representation of the ensemble, which we analyzed regarding base-stacking and -pairing.

While the GNRA conformation seems to be the main conformation of the loops, we also observed clusters of UNCG and other known tetraloop conformations within our obtained ensembles. Additionally, we used predicted structures from Farfar and AlphaFold and performed a comparison of all methods by determining the RMSE of the back-calculated observables, supporting the impression that the ensemble of the reweighted MD is in fact the most faithful representation of the RNA.

**What led you to study RNA or this aspect of RNA science?**

While my initial focus during my master's program was on organic synthesis, I had the opportunity of a fascinating internship in the Schwalbe Lab on NMR investigation of RNA and was instantly hooked to this field. During my master's thesis in the Schwalbe Lab, I learned more about the flexible constitution of RNA in solutions and continued working on it during my PhD, where I was able to work on the in silico approaches to it during cooperation with the Lindorff-Larsen Lab in addition to the NMR approach I was more familiar with.

**During the course of these experiments, were there any surprising results or particular difficulties that altered your thinking and subsequent focus?**

During the reweighting of the MD simulations, we had to decide which observables were to be used in the process and which to be used only later in the cross validation. We ran the reweighting therefore with different combinations of observables, and I was surprised how prone certain observables were to over-fitting while others, especially RDCs and J-couplings, were more resilient against this problem. Overall, I would say that I have become quite fond of RDCs as a set of observables, since they are reliably detectable and seem to enhance the understanding of the overall structure.

**If you were able to give one piece of advice to your younger self, what would that be?**

I would encourage my younger self to be more outgoing and work more closely with peers. While being self-reliant during studying is a good and important skill, there is much to be gained from the perspectives of others, and one is able to spot their own blind spots about a topic more easily when conversing about it.
